# An Oral Microencapsulated Vaccine Loaded by Sodium Alginate Effectively Enhances Protection Against GCRV Infection in Grass Carp (*Ctenopharyngodon idella*)

**DOI:** 10.3389/fimmu.2022.848958

**Published:** 2022-03-24

**Authors:** Chuang Xu, Meihua Qiao, Xingchen Huo, Zhiwei Liao, Jianguo Su

**Affiliations:** ^1^ Department of Aquatic Animal Medicine, College of Fisheries, Huazhong Agricultural University, Wuhan, China; ^2^ Laboratory for Marine Biology and Biotechnology, Pilot National Laboratory for Marine Science and Technology, Qingdao, China; ^3^ Hubei Hongshan Laboratory, Engineering Research Center of Green Development for Conventional Aquatic Biological Industry in the Yangtze River Economic Belt, Ministry of Education, Wuhan, China

**Keywords:** grass carp reovirus (GCRV), sodium alginate (SA), flagellin (FlaB), oral microencapsulated vaccine, grass carp

## Abstract

Grass carp reovirus (GCRV) is highly infectious and lethal to grass carp, causing huge economic losses to the aquaculture industry annually. Currently, vaccination is the most effective method against viral infections. Among the various vaccination methods, the oral vaccination is an ideal way in aquaculture. However, low protective efficiency is the major problem for oral vaccination owing to some reasons, such as antigen degradation and low immunogenicity. In our study, we screened the antigenic epitopes of GCRV-II and prepared an oral microencapsulated vaccine using sodium alginate (SA) as a carrier and flagellin B (FlaB) as an adjuvant, and evaluated its protective effects against GCRV-II infection in grass carp. The full length and three potential antigenic epitope regions of GCRV-II VP56 gene were expressed in *Escherichia coli* and purified by glutathione affinity column respectively. The optimal antigen (VP56-3) was screened by enzyme-linked immunosorbent assay (ELISA). Adjuvant FlaB was also expressed in *E. coli* and purified by Ni^2+^ affinity column. Subsequently, we prepared the oral vaccines using sodium alginate as a carrier. The vaccine (SA-VP56-3/FlaB) forms microsphere (1.24 ± 0.22 μm), examined by transmission electron microscopy, scanning electron microscopy, and dynamic light scattering assay. SA-VP56-3/FlaB vaccine has excellent stability, slow-release, and low toxicity by dynamic light scattering assay, release dynamic assay, *in vivo* fluorescence imaging system, hemolytic activity and cytotoxicity. Then we vaccinated grass carp orally with SA-VP56-3/FlaB and measured immune-related parameters (serum neutralizing antibody titer, serum enzyme activity (TSOD, LZM, C3), immune-related genes ((IgM, IFN1, MHC-II, CD8 in head kidney and spleen), IgZ in hindgut)). The results showed that SA-VP56-3/FlaB significantly induced strong immune responses, compared to other groups. The highest survival rate achieved in SA-VP56-3/FlaB microencapsulated vaccine (56%) in 2 weeks post GCRV challenge, while 10% for the control group. Meanwhile, the tissue virus load in survival grass carp is lowest in SA-VP56-3/FlaB group. These results indicated that SA-VP56-3/FlaB could be a candidate oral vaccine against GCRV-II infection in aquaculture.

## Highlights

1. VP56-3 had the strongest anti-serum binding capacity.
2. Oral vaccine increased protection by 46%.
3. Oral vaccine can slow-release and resistant to intestinal protease action.

## 1 Introduction

Grass carp (*Ctenopharyngodon idella*) is one of the most economically important cultured fish in China (1, 2). However, grass carp reovirus (GCRV) has seriously harmed the grass carp cultivation industry in China due to its high infectiousness and high mortality rate (3, 4). GCRV belongs to the genus Aquareovirus and was the first strain of aquatic animal virus identified in China (5). It is a double-stranded RNA virus with a structure of 20-sided symmetrical spherical particles with a diameter of 70-80 nm and the genome of GCRV has 11 segments (6). According to previous studies, GCRV is divided into three main genotypes (GCRV-I, GCRV-II, and GCRV-III), and GCRV-II is capable of causing hemorrhagic disease with a high mortality rate in grass carp, which has caused great harm to the global grass carp cultivation industry (7, 8).

Vaccination is one of the effective strategies to protect fish from viral diseases (9). In recent years, with the development of science and technology, new vaccines against GCRV infection have been developed very rapidly (1, 10). Among these new vaccines, both genetically engineered vaccines and subunit vaccines have been reported to be effective in protecting grass carp from GCRV infection under experimental conditions (11, 12). However, for fish, the injectable immunization method cannot be applied on a large scale in farms due to the high cost and handling stress that may cause the mortality of vaccinated fish (13). Therefore, the development of cost-effective vaccines that are easily applicable at the farm level is an issue of growing importance (14).

The mucosal surface constitutes the largest body surface area of fish in constant contact with the external environment, and the skin, gills, nose and intestine of fish contain a variety of immune factors and immune cells that protect against pathogen invasion, and they are responsible for maintaining immunological homeostasis (15, 16). Vaccination *via* the mucosal route of administration reduces the amount of antigen administered and is less stressful than the injectable route of administration (15). The use of mucosal vaccination not only induces local innate and adaptive immune responses in fish, but also produces protective systemic immune responses (17). Oral administration with natural and noninvasive factors is a conceptually simple approach to delivering vaccines in animals, particularly fish. Oral subunit vaccines are one of the desirable vaccination strategies for fish because of their stress-free handling, convenience, cost-effectiveness, and ease of application for mass vaccination (18, 19). Oral administration can effectively mimic the natural feeding process of fish by delivering the target antigen directly to the digestive tract, inducing a specific mucosal immune system in fish, activating the mucosal immune barrier, and also inducing a specific immune response from the lymphatic system to resist the virus when it passes through the mucosa (20–22). However, the drawback of the low immunogenicity and antigens degradation of oral subunit vaccines needs to be addressed (23).

Screening for antigens is an effective method to improve immunogenicity (24, 25). Most of the available studies have shown that antigen epitope screening can significantly improve the immune effect of virus suppression (24–26). VP56 is a protein encoded by the S7 fragment of GCRV-II with a structure similar to the outer fibrillar protrusion protein of adenovirus. It is speculated that VP56 is an adsorption protein on the surface of GCRV-II that can bind to receptors on the host cell membrane and may play a crucial role in the process of virus invasion (27, 28), and it has been shown to have good immunogenicity and can produce neutralizing antibodies (29). The use of adjuvants that enhance the immune response in the mucosal immune compartment will greatly enhance the ability of oral subunit vaccines to induce humoral immune responses (30). A specific group of immune enhancers that can be used as mucosal adjuvants is toll-like receptors (TLRs) (31). TLRs are pattern recognition receptors (PRRs) that specifically recognize agonists of their cognate family, also known as microbe-associated molecular patterns (32). Flagellin B (FlaB), an agonist of TLR5, has been reported to be a widely used mucosal immune adjuvant in mammals (33). However, at present, the use of FlaB as an adjuvant for viral vaccines in aquaculture has been rarely reported.

To ensure that the antigen and adjuvant can work *in vivo*, it is necessary to overcome the difficulty that the protein is difficult to resist hydrolysis by digestive tract proteases and deactivation (34). Sodium alginate is a natural polysaccharide that protects protein-based antigens from the pH environment and proteases in the stomach and intestines and has mucoadhesive properties that allow it to adhere to mucosal tissues and prolong the residence time of the drug (14, 34, 35). In recent years, the use of sodium alginate as an antigen delivery vehicle is a promising antigen delivery method for the development of oral vaccines, which has attracted much attention in the field, and many studies have shown that oral vaccines using sodium alginate as a carrier have achieved better protection in different fish and mammals (14, 36, 37).

In this study, we investigated the protective effect of an oral microencapsulated vaccine based on grass carp reovirus (GCRV-II) VP56 protein and adjuvant FlaB delivered by sodium alginate against GCRV-II infection to develop a handling stress-free oral vaccine that is easily applicable for mass vaccination of fish with GCRV-II at farm level.

## 2 Materials and Methods

### 2.1 Virus, Bacteria, and Fish

Grass carp reovirus (GCRV-II) used in this experiment is GCRV 097, stored in our laboratory. GCRV 097 was propagated in *C. idella* kidney (CIK) cells at 28°C with 5% CO_2_, in DMEM (12100046, Gibco, Beijing, China) medium supplemented with 10% FBS (10100147, Gibco, Beijing, China), 100 U/mL penicillin (I9532, Sigma, Shanghai, China), and 100 U/mL streptomycin (85886, Sigma, Shanghai, China).

The *Escherichia coli* (DH5a or DE3plys) (Invitrogen, Carlsbad, CA, USA) used to express the recombinant protein is stored in our lab in glycerol at -80°C.

Healthy grass carp (GCRV-II free; weight about 15 g) obtained from the Xingfu Farm (Huang gang, China) were maintained in fiberglass tanks (300 L of water) at 28 ± 1°C. The grass carp were fed with commercial feed (04047, Haid, Shenzhen, China) once a day (12:00 am), and after two weeks of temporary rearing, the grass carp were divided into 15 tanks with 50 fish per tank. The experiment was divided into five groups, including the control group, SA group, SA-FlaB group, SA-VP56-3 group, SA-VP56-3/FlaB group, each group had three parallel, total of 150 fish (50 × 3), each group of fish was fed once a day for 28 days, feeding rate is 3% of fish weight. Feeding normal feed after the viral challenge. All animal experiments described comply with the National Guidelines for the Care and Use of Laboratory Animals (CNAS-CL06:2018) and were approved by the Committee on the Ethics of Animal Experiments of Huazhong Agricultural University, China (HZAUFI-2021-0028).

### 2.2 Gene Cloning and Analysis

The antigenic epitopes and hydrophilicity of VP56 (GenBank: MK675081.1) were analyzed by the DNA Star program, and the full length of VP56 was divided into three fragments, VP56-1 (0-420 bp), VP56-2 (421-939 bp), and VP56-3 (940-1461 bp).

Total RNA of GCRV 097 (GCRV-II) was extracted with RNAiso Plus kit (TaKaRa), the purification and concentration were measured by a NanoDrop 2000 spectrophotometer (Thermo Scientific, USA), and the quality was evaluated using 1.5% agarose gel electrophoresis. mRNAs were reverse-transcribed into cDNAs respectively with MMLV reverse transcriptase, RNase inhibitor (Thermo Fisher Scientific, USA), hexamer random primer. The primers for VP56-1, VP56-2, VP56-3, VP56 were listed in **Supplementary Table S1**. The genes were amplified on a ProFlex™ PCR system with the following conditions: 95°C for 5 min, followed by 35 cycles at 95°C for 30 s, 56°C for 30 s, 72°C for 1 min, and the final extension at 72°C for 10 min. The PCR product was purified using a DNA gel purification kit (Axygen, Hangzhou, China) and subsequently were cloned into the pGEX-4T-1 expression vector (Novagen) after digestion using a combination of *Bam*H I and *Xho* I (New England Biolabs, USA). The recombinant plasmid (pGEX-4T-1-VP56-1, pGEX-4T-1-VP56-2, pGEX-4T-1-VP56-3, pGEX-4T-1-VP56) was transformed into *E. coli* (DH5a) and positive clones were selected by PCR and sequenced by QingKe Bioscience and Technology Company (Wuhan, China).

### 2.3 Recombinant Expression, Purification, and Immunological Protein Screening

#### 2.3.1 Recombinant Expression and Purification of VP56 Full-Length and Fragment Proteins

The recombinant expression plasmids (pGEX-4T-1-VP56-1, pGEX-4T-1-VP56-2, pGEX-4T-1-VP56-3, pGEX-4T-1-VP56) were transformed into *E. coli* (DE3plys) and incubated in LB medium at 37°C for 4 h. Then IPTG (1 mmol/L) was added to induce expression at 37°C for 4 h.

The bacteria were collected by centrifugation (25°C, 5000 rpm, 10 min). The bacteria was resuspended with PBS (pH = 7.4) and pulverized with a high-pressure pulverizer (880 MPa). The supernatant and precipitate were collected by centrifugation (4°C, 12000 rpm, 1 h). Purification of recombinant protein (VP56-1, VP56-2, VP56-3, VP56) according to the previous method (38). Briefly, the proteins were bound to 10 mL glutathione beads (Smart-Lifesciences, China), eluted through a gradient of buffer B (50 mM Phosphate Buffer, 10 mM L-Glutathione reduced (pH = 8.0, 25°C)) and dialysis in the 50 mM Phosphate Buffer (pH = 8.0, 25°C). Validation of purified recombinant proteins using SDS-PAGE (Solarbio) and quantification of recombinant proteins using BCA kits (Solarbio).

#### 2.3.2 Screening for Potential Antigens

The grass carp were randomly divided into 5 groups, including the VP56-1 group, VP56-2 group, VP56-3 group, VP56 group, and control group (10 fish in each group). Each group of grass carp was injected intraperitoneally with an equal amount of protein (100 μL, 100 μg/mL), while the control group was injected with PBS (pH = 7.4, 100 μL). After three weeks, five fish from each group were anesthetized in MS-222, and blood was collected. The blood was placed at 4°C overnight, then serum was collected by centrifugation (4°C, 3500 rpm, 30 min) and stored at -80°C.

Antigen binding capacity according to the previous method (24). Briefly, the ELISA plate was coated by the diluted anti GCRV-II serum (1:500, 1:1000, 1:2000, 1:4000, 1:8000) at 4°C for one day, followed by incubation with 1% bovine serum albumin (BSA) in PBS (pH = 7.4) for 1 h at 37°C to prevent non-specific binding. The well was washed by PBST and incubated with VP56-1, VP56-2, VP56-3, VP56 proteins, and PBS (pH = 7.4). Afterward, it was incubated with GST-tag mAb (1:2500), and HRP-labeled sheep anti-mouse IgG mAb (1:2500) was incubated with the well, adding with TMB (Tiangen Biotech, Beijing, China) substrate. The color reaction was stopped by 2M H_2_SO_4_ and read at 450 nm of wavelength.

Neutralizing antibodies were determined according to the previous method (39). Briefly, the serum of each group was heat-inactivated at 45°C for 30 min, the diluted serum (1:1, 1:10, 1:20, 1:40) was mixed with an equal volume of GCRV-II containing 50% TCID_50_/mL and incubated at 37°C for 1 h. We added 100 µL of the diluted serum mixture to each well containing CIK cells and incubated the plate at 28°C for 1 h. Then, the mixtures were gently aspirated, and 0.2 mL of fresh DMEM supplemented with 2% FBS was added back to each well. The 96-well microplates were incubated at 28°C for 5-7 days. Negative serum was used as the control group. Survival rate determination of CIK cells by the 3-(4,5-dimethylthiazol-2-yl)-2,5-diphenyltetrazole (MTT) assay. Briefly, the cell culture medium was replaced with 10% MTT solution, and the cells were incubated at 37°C and 5% CO_2_ for 4 h. The absorbance at 595 nm was measured using a microplate reader (Spectra Max M5, Molecular Devices, Sunnyvale, CA, USA).

### 2.4 Preparation of Oral Microencapsulated Vaccine

#### 2.4.1 SDS-PAGE Analysis and Western Blot (WB)

Recombinant protein VP56-3 and adjuvant protein FlaB (preserved in our lab) were verified by 10% SDS-PAGE and WB. The protein samples (10 μL) were separated by 10% SDS-PAGE and the gels were stained using Coomassie Brilliant Blue G-250 (C.I.42655, Sigma, Shanghai, China). In addition, the gel was transferred to the NC membrane at 9 V and the membrane was closed using 5% skim milk powder diluted in TBST for 2 h. The membrane was washed three times with TBST, incubated with GST-tag mAb (VP56-3) or His-tag mAb (FlaB) diluted 1:2500 for 2 h, washed four times with TBST, diluted 1:2500 with HRP-labeled sheep anti-mouse IgG mAb was incubated with the membrane for 45 min, TBST washed three times, subsequently stained with Clarity TM Western ECL Substrate (Bio-Rad), and finally imaged by the Amersham Imager 600 (Little Chalfont).

#### 2.4.2 Preparation and Characterization of Oral Vaccine

We prepared the oral vaccine SA-VP56-3/FlaB using the encapsulation method. Briefly, 1.6 mg/mL of VP56-3 and 0.4 mg/mL of FlaB were mixed well and then mixed thoroughly with 1.5% sodium alginate (Control was prepared by replacing the protein with PBS pH = 7.4). The mixed solution was slowly added dropwise at 4:6 to paraffin (containing emulsifier Span-80), stirred at 1500 rpm/min for 30 min. After full emulsification, the 8% (M/V) CaCl_2_ was added to the emulsified sodium alginate protein mixture to calcify and form microspheres, the precipitate was collected by centrifugation (25°C, 8000 rpm, 5 min) and washed three times with 0.01 mol/L CH₃COONa (pH = 4.0). Then stirred with 0.8% chitosan (pH = 5.0) at 1500 r/min for 15 min, the precipitate was collected by centrifugation (25°C, 8000 rpm, 5 min), washed three times, and stored in lyophilization.

Centrifuge 5 mL of SA-VP56-3/FlaB suspension, add SA-VP56-3/FlaB precipitate into the appropriate amount of PBS (pH = 7.4), ultrasonically break it up, and dissolve it fully. The supernatant was collected by centrifugation (4°C, 12000 rpm, 30 min). The protein concentration was quantified by BCA kit, and the encapsulation efficiency and loading rate were calculated according to the formula:


(1) EE=A/B×100%

(2) LE=A/C×100%

Where A denotes the amount of protein in the actual SA-VP56-3/FlaB, B denotes the amount of protein in the theoretical SA-VP56-3/FlaB, and C denotes the total mass of SA-VP56-3/FlaB (n = 3).

The formed SA-VP56-3/FlaB were resuspended in distilled water. The particle size distribution and zeta potential of SA-VP56-3/FlaB were determined by dynamic light scattering using a Malvern Nano-ZS 90 laser particle size analyzer (Malvern Instruments, UK) at a detector angle of 90°, 670 nm, and temperature of 25°C. The morphology of SA-VP56-3/FlaB was observed by transmission electron microscopy (TEM, SPA-400, Japan) and scanning electron microscope (SEM, Model JSM-6390/LV, NTC, Japan).

#### 2.4.3 Hemolysis and Cytotoxicity Tests

A hemolysis assay was performed with grass carp erythrocytes. Briefly, 3 mL of fresh peripheral blood was collected using sodium heparin tubes, and erythrocytes were collected by centrifugation (4°C, 500 g, 10 min). Flushing with PBS (pH = 7.4) and resuspending. Added 100 μL SA-VP56-3/FlaB and erythrocytes (100 μL) to a 96-well plate and incubate at 37°C for 2 h. After centrifugation (4°C, 500 g, 10 min), the absorbance of each well at 450 nm was measured using a microplate reader.

The cytotoxicity of SA-VP56-3/FlaB to CIK cells was measured by the MTT assay. Cells were inoculated into 96-well plates (1.0 × 10^4^ per well), and then different concentrations of SA-VP56-3/FlaB, SA, and VP56-3/FlaB were added. After co-culture at 37°C for 1 h, the cell culture medium was replaced with 10% MTT solution. The cells were incubated at 37°C and 5% CO_2_ for 4 h. The absorbance at 595 nm was measured using a microplate reader.

#### 2.4.4 Release Rate and Intestinal Fluorescence

The release of VP56-3/FlaB from SA-VP56-3/FlaB was performed according to the previous description (40). In release medium (PBS pH = 7.4 or Grass carp intestinal fluid), *in vitro* release of SA-VP56-3/FlaB was detected at 25°C for 36 h. 1 mL SA-VP56-3/FlaB (1 mg/mL) were placed into EP tubes with 1 mL PBS or intestinal fluid in a shaking incubator (ZQPL-200, Tianjin, China) at 100 rpm for 36 h at 25°C. The mixture was tested by a BCA Protein Assay Kit (PC0020, Solarbio, Beijing, China) at specified time intervals (0 h, 6 h, 12 h, 18 h, 24 h, 30 h, 36 h). The amount of protein in the supernatant at each time point was counted to produce an *in vitro* release curve.

SA-VP56-3/FlaB were prepared by FITC-VP56-3 and FITC-FlaB under light-proof conditions. 5 mg SA-VP56-3/FlaB was resuspended to 5 mL PBS (pH = 7.4), and stored under light-proof conditions. 27 grass carp were randomly divided into 3 groups, including the control group, FITC-VP56-3/FlaB group, SA- FITC-VP56-3/FlaB group, and gavaged with 200 μL samples at 0 h, 6 h and 12 h (three grass carp each time). The control group was gavaged with 200 μL PBS (pH = 7.4). Grass carp were dissected to expose the abdominal cavity at 12 h, and the fluorescence in the intestine was analyzed using a small animal *in vivo* optical imaging system.

### 2.5 Oral immunization and Viral Challenge

To evaluate the protective effect of SA-VP56-3/FlaB, we conducted a viral challenge experiment on grass carp. Briefly, grass carp were divided into five groups (n = 150). The feeding situations before challenge were as follows: common fodder (Control group), SA (100 mg/kg, SA group), SA-FlaB (50 mg/kg, SA-FlaB group), SA-VP56-3 (150 mg/kg, SA-VP56-3 group), SA-VP56-3/FlaB (200 mg/kg, SA-VP56-3/FlaB group). Each group of fish was fed once a day for 28 days. Stopped feeding for one day before the challenge, and gave common fodder after challenge. Each group of grass carp was injected intraperitoneally with 10^7^ TCID_50_/mL of 100 μL GCRV 097 on D29, and the mortality of each group was recorded and monitored daily. Blood, head kidney, spleen, and hindgut were taken from each group of grass carp on D0, D28, and D40.

On D42, the viral load was measured by taking the head kidney, spleen, and hindgut of each group of grass carp (n = 5). Total RNAs of head kidney, spleen, and hindgut were extracted with RNAiso Plus kit, the purification and concentration were measured by a NanoDrop 2000 spectrophotometer (Thermo Scientific, USA), and the quality was evaluated using 1.5% agarose gel electrophoresis. mRNAs were reverse-transcribed into cDNAs respectively with MMLV reverse transcriptase, RNase inhibitor (Thermo Fisher Scientific, USA), hexamer random primer. The viral protein VP56 was measured by qRT-PCR using the primers VP56-Q (**Supplementary Table S1**). 18S rRNA (GenBank: EU047719.1) was used as the control gene.

### 2.6 Enzyme Activity Assay in Serum and Quantitative RT-PCR Analysis of Immune-Related Gene Expression

#### 2.6.1 Enzyme Activity Assay in Serum

We collected blood from each group of grass carp according to time points (D0, D28, D40). Fresh blood samples were placed overnight at 4°C. After centrifugation (4°C, 3500 rpm, 30 min), the serum was collected for enzyme activity detection, and the remaining serum was stored at -80°C. Serum enzyme activities including total superoxide dismutase (TSOD), lysozyme (LZM), complement 3 (C3) were measured as a previous report (41). These three indicators were determined by kits purchased from Nanjing Jiancheng Institute of Biological Engineering.

#### 2.6.2 Quantitative RT-PCR Analysis of Immune-Related Gene Expression

We collected the head kidney, spleen, and hindgut of each group of grass carp according to time points (D0, D28, D40). Total RNAs of head kidney, spleen, and hindgut were extracted with RNAiso Plus kit and reverse transcribed into cDNAs according to the method in 2.5. The primers for qRT-PCR analyses were listed in **Supplementary Table S1**. Sequence source (GenBank): 18S rRNA (EU047719.1), IFN1 (AB196166.1), MHC-II (JF436931.1), CD8 (GQ355586.1), IgM (DQ417927.1), IgZ (DQ489733.1). 18S rRNA was used as a reference control gene, and the relative mRNA expression levels were calculated with the CT method (2^-△△CT^).

### 2.7 Determination of Serum Neutralizing Antibody Levels and Antigen Binding Capacity

Serum was collected from each group of grass carp on D28, and the dilution ratio was 1:500, 1:1000, 1:2000, 1:4000, 1:8000. Antigen binding capacity was determined for each group of grass carp serum as described in method 2.3.2.

Serum was collected from each group of grass carp on D28, and the dilution ratio was 1:1, 1:10, 1:20, 1:40. Neutralizing antibody level was determined for each group of grass carp serum as described in method 2.3.2.

### 2.8 Statistical Analysis

The results were presented as means ± standard deviation (SD) and all the statistical analyses were done using SPSS 26.0 package. The experimental data were subjected to the Kruskal-Wallis test, followed by Dunn’s multiple comparison (with Bonferroni adjustment) to identify the significance (p < 0.05). Different superscript letters in each group (a, b, c, and d) denote significant variations. The protection rates were analyzed by Mantel-Cox test, * denotes significant variation.

## 3 Results

### 3.1 VP56-3 Is the Optimal Antigenic Epitope Fragment

**Figure 1:**
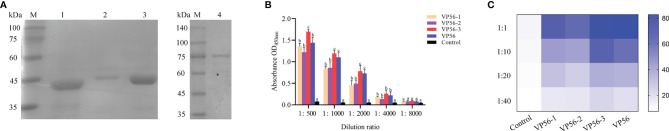
Recombinant expression and screening of VP56 full-length and fragment protein. **(A)** SDS-PAGE analyses of VP56 full-length and fragment protein. Lane M: protein marker; Lane 1: Purified VP56-1; Lane 2: Purified VP56-2; Lane 3: Purified VP56-3. Lane 4: Purified VP56. **(B)** ELISA assays were used to screen full-length and fragment proteins to identify those with the highest binding capacity to anti GCRV-II serum. **(C)** Verification of the inhibitory ability of each protein serum against GCRV-II by neutralizing antibody assay. Data are expressed as mean ± SD (n = 4). Different lowercase letters in each group (a, b and c) denote significant variations suggested by the Kruskal-Wallis statistics followed by the Dunn's multiple comparison (p < 0.05).

The VP56 (1539 bp) gene, VP56-1 (420 bp) fragment, VP56-2 (522 bp) fragment, and VP56-3 (525 bp) fragment were cloned into the prokaryotic expression plasmid pGEX-4T-1, and *E. coli* was induced by IPTG to express proteins of suitable size (VP56 about 82 kDa, VP56-1 about 41 kDa, VP56-2 about 44 kDa, VP56-3 about 44 kDa), and the size of the recombinant proteins was verified by SDS-PAGE (**Figure 1A**). The binding ability of VP56-1, VP56-2, VP56-3, and VP56 proteins to anti GCRV-II grass carp serum was screened by ELISA assay. The results showed that the binding ability of the VP56-3 fragment was the strongest, followed by VP56 full-length protein, which was significantly different compared with VP56-1 and VP56-2. When the anti GCRV-II grass carp serum dilution ratio was 1:4000, VP56-3 could still bind to serum, and when the serum dilution ratio was 1:8000, each protein was barely bound to serum (**Figure 1B**). The inhibitory effect of VP56-1, VP56-2, VP56-3, and VP56 serum on GCRV-II was analyzed by neutralizing antibody assay. The results showed that VP56-3 had the strongest inhibitory effect on GCRV-II, followed by VP56. When the serum dilution of VP56-3 and VP56 was 1:40, it was still can inhibit GCRV-II, and the cell survival rate was about 20%, while the negative serum could barely inhibit the virus (**Figure 1C**). The results of the neutralizing antibody assay were generally consistent with those of the ELISA assay, and these results suggested that VP56-3 is a potentially valuable antigenic epitope for GCRV-II.

### 3.2 Oral Vaccine Is Stable, Slow-Release, Low Toxicity, and Resistant to Intestinal Enzymatic Digestion

**Figure 2:**
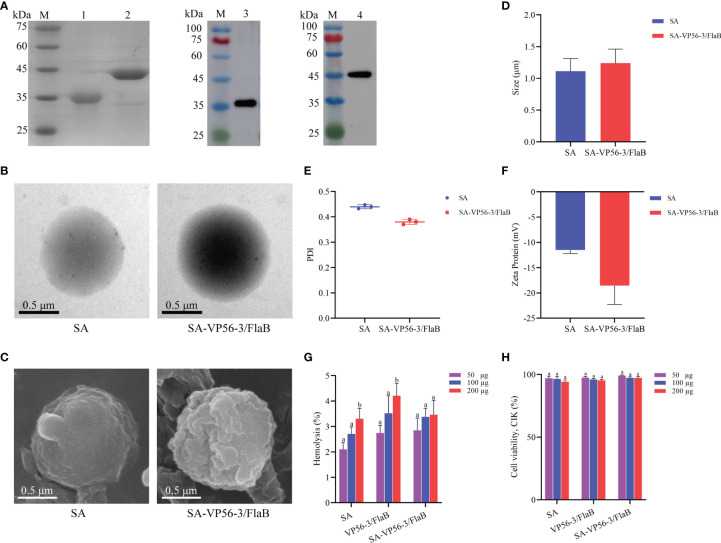
Recombinant expression of VP56-3 and FlaB, preparation and biosafety assay of the oral microencapsulated vaccine. **(A)** SDS-PAGE and WB analyses of VP56-3 and FlaB. Lane M: protein marker; Lane 1: Purified FlaB; Lane 2: Purified VP56-3; Lane 3: FlaB was confirmed by WB with His-tag mAb; Lane 4: VP56-3 was confirmed by WB with GST-tag mAb. **(B)** Image of SA under TEM (left); Image of SA-VP56-3/FlaB under TEM (right). **(C)** Image of SA under SEM (left); Image of SA-VP56-3/FlaB under SEM (right). **(D)** Nanoparticle size analysis. **(E)** PDI distribution coefficient. **(F)** Zeta potential analysis. **(G)** Hemolysis study of SA, VP56-3/FlaB, and SA-VP56-3/FlaB. Hemolytic activity of each group was detected with 2% grass carp erythrocytes for 1 h at 37 °C. Data are expressed as mean ± SD (n = 4). **(H)** Toxicity effect of SA, VP56-3/FlaB, and SA-VP56-3/FlaB on CIK cells. MTT method was used for determination. Data are expressed as mean ± SD (n = 4). Different lowercase letters in each group (a and b) denote significant variations suggested by the Kruskal-Wallis statistics followed by the Dunn’s multiple comparison (p < 0.05).

**Figure 3:**
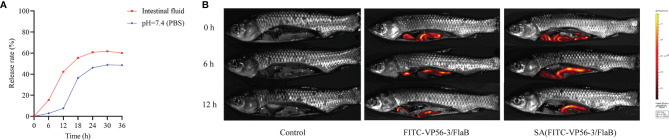
*In vitro* release rate and intestinal imaging of gavage SA (FITC-VP56-3/FlaB). **(A)** Release rate of SA-VP56-3/FlaB in PBS (PH = 7.4) or grass carp intestinal fluid over 36h. **(B)** Fluorescence of control in the intestine of grass carp with 0 h, 6 h, 12 h (left); Fluorescence of FITC-VP56-3/FlaB in the intestine of grass carp with 0 h, 6 h, 12 h (mid); Fluorescence of SA (FITC-VP56-3/FlaB) in the intestine of grass carp with 0 h, 6 h, 12 h (right).

The VP56-3 protein (44 kDa) and the adjuvant protein FlaB (34 kDa) were verified by SDS-PAGE and WB (**Figure 2A**). Oral vaccine SA-VP56-3/FlaB was prepared according to the embedding method, and the use of sodium alginate as an oral vaccine delivery vehicle to encapsulate antigen and adjuvant proteins. Under transmission electron microscopy (TEM), the unencapsulated SA was spherical in PBS (pH = 7.4), and when proteins are encapsulated in SA, a darker color of protein was observed inside SA-VP56-3/FlaB (**Figure 2B**). Under scanning electron microscopy (SEM), the surface of the unencapsulated SA was smoother, while SA-VP56-3/FlaB had irregular protrusions on the surface (**Figure 2C**). The physical characterization of SA-VP56-3/FlaB was measured using dynamic light scattering and showed that the mean particle size of the unencapsulated SA was 1.18 ± 0.39 μm, the zeta potential was -12.40 ± 0.25 mV, and the PDI was 0.42 ± 0.01 (PDI < 0.5), and after encapsulation of proteins, the mean particle size of SA-VP56-3/FlaB increased to 1.24 ± 0.22 μm, the zeta potential increased to -18.60 ± 0.47 mV and the PDI was 0.38 ± 0.01 (PDI<0.5) (**Figures 2D–F**). The EE and LE of SA-VP56-3/FlaB were 69.6 ± 3.12% and 16.4 ± 1.95%, respectively (**Table 1**). SA-VP56-3/FlaB did not show significant cytotoxicity and hemolysis by hemolysis assay and MTT assay in the range of 50-200 μg (**Figures 2G, H**). By drug release analysis, VP56-3/FlaB was released from SA-VP56-3/FlaB in different solutions (PBS pH = 7.4 or intestinal fluid). Release of VP56-3/FlaB from SA-VP56-3/FlaB was faster in intestinal fluid from 0-24 h compared to PBS (pH = 7.4), with a cumulative release rate of 60% (**Figure 3A**). By intestinal fluorescence analysis, SA-VP56-3/FlaB can remain in the intestine of grass carp for a longer period compared to naked VP56-3/FlaB during 0-12 h (**Figure 3B**). These results suggested that SA-VP56-3/FlaB has a spherical structure, stability, slow-release, low toxicity, and is resistant to hydrolysis by grass carp intestinal proteases.

**Table 1:** Properties of SA-VP56-3/FlaB and SA (mean ± SD, n = 3).

Name	Mean particle size (μm)	PDI	Zeta potential (mV)	EE (%)	LE (%)
SA-VP56-3/FlaB	1.24 ± 0.22	0.38 ± 0.01	-18.60 ± 0.47	69.6 ± 3.1	16.4 ± 1.95
SA	1.18 ± 0.39	0.42±0.01	-12.40 ± 0.25	N/A	N/A

### 3.3 Immune Protection Effect of Oral Vaccine

**Figure 4:**
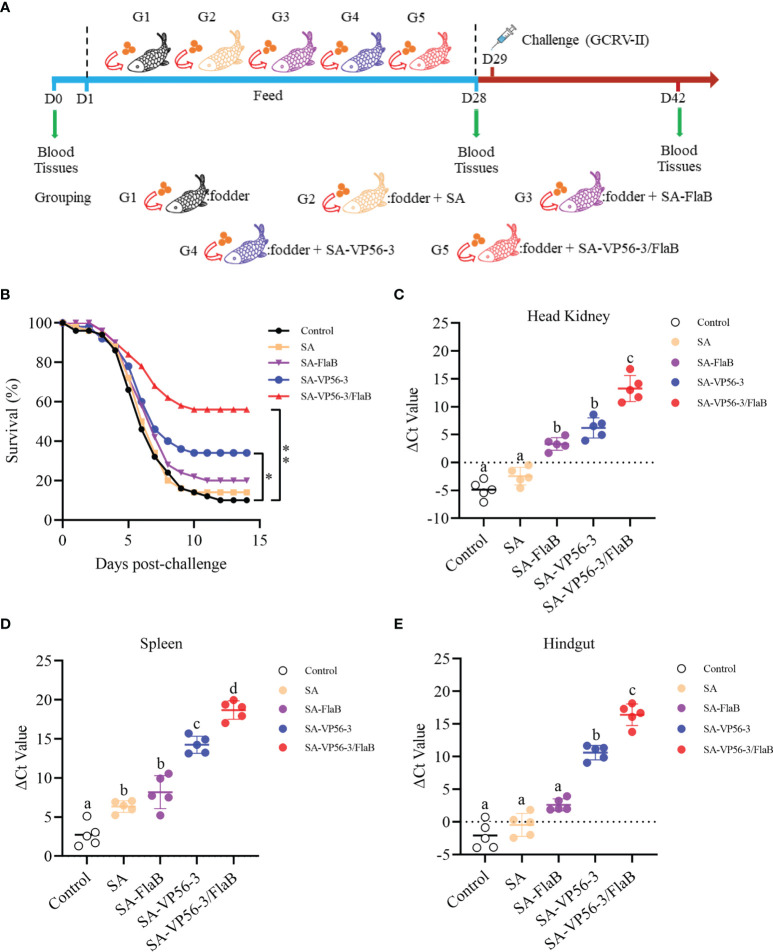
Protection rate of SA-VP56-3/FlaB against GCRV-II (10^7^TCID_50_/mL) infection and tissues viral load. **(A)** Schematic of the oral immunization, GCRV-II challenge, and sampling. **(B)** Survival of grass carp (n = 50) was monitored and calculated within 14 days after the viral challenge. **(C)** Head kidney viral load of surviving grass carp on D42. **(D)** Spleen viral load of surviving grass carp on D42. **(E)** Hindgut viral load of surviving grass carp on D42. ΔCT indicates the difference between CT_vp56_ and CT_18S rRNA_. Data are expressed as mean ± SD (n = 5). Different lowercase letters in each group (a, b, c and d) denote significant variations suggested by the Kruskal-Wallis statistics followed by the Dunn’s multiple comparison (p < 0.05).

Grass carp were challenged with 100 μL of GCRV 097 (10^7^ TCID_50_/mL) injected intraperitoneally and survival was recorded daily for two weeks (**Figure 4A**). All groups showed dramatic mortality from D4 and reached a steady state on D11. Two weeks later, the SA-VP56/FlaB group had the highest survival rate of 56%, the survival rates of the other groups (SA-VP56, SA-FlaB, SA) were 34%, 22%, and 12% respectively. The survival rate in the control group was only 10% (**Figure 4B**). We also determined the viral load of GCRV-II in the head kidney, spleen, and hindgut of surviving individuals. The results showed that the SA-VP56-3/FlaB group had the lowest viral load, which was significantly different from the other groups, and followed by the SA-VP56 group which was significantly different compared to the control group. Of the three tissues, the highest viral load was found in the head kidney, followed by the intestine, and the lowest viral load was found in the spleen (**Figures 4C–E**). These results suggested that SA-VP56-3/FlaB can alleviate tissue viral load and effectively protect grass carp from GCRV-II infection.

### 3.4 Serum Innate Immune Indices

**Figure 5:**
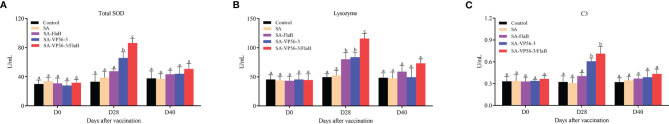
Effect of SA-VP56-3/FlaB on nonspecific immune parameters. **(A)** Serum TSOD activity. **(B)** Serum LZM activity. **(C)** Serum C3 activity, determined on D0, D28, D40. Data are expressed as mean ± SD (n = 4). Different lowercase letters in each group (a, b and c) denote significant variations suggested by the Kruskal-Wallis statistics followed by the Dunn's multiple comparison (p < 0.05).

The levels of TSOD, LZM, and C3 were measured in different immunization groups (SA-VP56-3/FlaB group, SA-VP56-3 group, SA-FlaB group, SA group) and control group after oral immunization and viral challenge. On D28, the highest TSOD and LZM levels were found in the SA-VP56-3/FlaB group, which was significantly different from the other groups (**Figures 5A, B**). The SA-VP56-3/FlaB group and SA-VP56-3 had the highest C3 level, which was significantly different from the other groups (**Figure 5C**). The SA-VP56-3 group had somewhat elevated levels of TSOD and LZM, which were significantly different compared to the control group (**Figures 5A, B**). The SA- FlaB group had a somewhat elevated level of LZM, which was significantly different compared to the control group (**Figure 5B**). The TSOD, LZM, and C3 contents of the control group and the SA group were almost indistinguishable. On D40, the TSOD, LZM, and C3 levels converged to normal levels, there was little significant difference between the groups (**Figures 5A–C**). These results suggested that SA-VP56-3/FlaB can enhance the non-specific immune system of grass carp.

### 3.5 Oral Vaccine Upregulates the Expression Levels of Immunity-Related Genes

**Figure 6:**
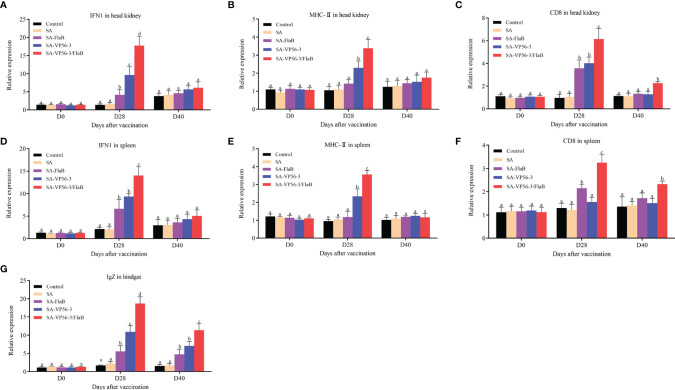
Transcriptional response of immune-related genes in head kidney, spleen, and hindgut of grass carp after oral immunization and viral challenge. Total RNAs were extracted from the head kidney, spleens, and hindgut on D0, D28, D40, and the transcription levels of immune-related genes. **(A–C)** The expression levels of immune-related genes (IFN1, MHC-II, CD8) mRNA in head kidney. **(D–F)** The expression levels of immune-related genes (IFN1, MHC-II, CD8) mRNA in spleen. **(G)** The expression level of IgZ mRNA in hindgut. The 18S rRNA gene was used as the control gene. Data are expressed as mean ± SD (n = 4). Different lowercase letters in each group (a, b, c and d) denote significant variations suggested by the Kruskal-Wallis statistics followed by the Dunn’s multiple comparison (p < 0.05).

We detected the head kidney and spleen immune-related genes (IFN1, CD8, MHC-II) and hindgut IgZ genes by qRT-PCR after oral immunization and viral challenge. The results showed that the expression levels of IFN1, CD8, MHC-II, and IgZ mRNA in the SA-VP56-3/FlaB group were significantly upregulated on D28, which was significantly different compared with the other groups (**Figures 6A–G**). On D40, the expression levels of IFN1 and MHC-II gene mRNA converged to normal levels, while CD8 and IgZ mRNA expression in the SA-VP56-3/FlaB group remained at a high level and were significantly different from the other groups (**Figures 6A–G**). These results suggested that SA-VP56-3/FlaB can effectively induce specific immune responses in grass carp.

### 3.6 Oral Vaccine Enhances Serum Antibody Production

**Figure 7:**
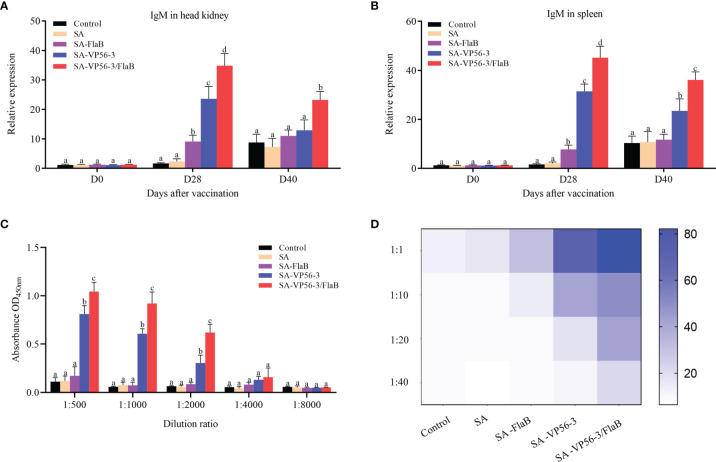
Expression levels of IgM mRNA in head kidney and spleen of grass carp, serum neutralizing antibody levels, and antigen binding capacity. **(A)** IgM mRNA expression level in head kidney on D0, D28, D42. **(B)** IgM mRNA expression level in spleen on D0, D28, D40. **(C)** ELISA assay to determine the binding ability of each group of serum to specific antigens on D28. **(D)** Neutralizing antibody assay to determine the inhibitory ability of each group of serum against GCRV-II on D28. Data are expressed as mean ± SD (n = 4). Different lowercase letters in each group (a, b, c and d) denote significant variations suggested by the Kruskal-Wallis statistics followed by the Dunn’s multiple comparison (p < 0.05).

To compare the antibody levels in the different immunization groups (SA-VP56-3/FlaB group, SA-VP56-3 group, SA-FlaB group, SA group) and the control group. We measured the expression levels of IgM mRNA in the head kidney and spleen after oral immunization and viral challenge by qRT-PCR. The results showed that the SA-VP56-3/FlaB group had the highest expression levels of IgM mRNA, which was significantly different from the other groups, and SA-VP56-3/FlaB induces higher levels of IgM mRNA than D40 at D28 (**Figures 7A, B**). We assessed the ability of D28 serum samples to inhibit GCRV-II using a neutralizing antibody assay. The results showed that the SA-VP56-3/FlaB group serum was the most potent inhibitor of GCRV-II. When the serum was diluted at 1:1, the cells survival rate of 82% in the SA-VP56-3/FlaB group, which was significantly different from the other groups, and the survival rates of the SA-VP56-3 group, SA-FlaB group, SA group, control group were 50%, 26%, 16%, 15%, respectively. When the serum was diluted at 1:40, the cell survival rate in the SA-VP56-3/FlaB group was 28%, whereas in the other groups the cell survival rate was only 8% (**Figure 7D**). We analyzed the specific binding ability of each group of serum to the antigen by ELISA. The results showed that the serum from the SA-VP56-3/FlaB group had the strongest ability to bind to the VP56-3 antigen, followed by the SA-VP56-3 group while the serum from the other groups had almost no ability to bind to the VP56-3 antigen, and when the serum was diluted 1:4000, the serum from all groups barely bound to the VP56-3 antigen (**Figure 7C**). These results suggested that the SA-VP56-3/FlaB group can induce a stronger humoral immune response in grass carp.

## 4 Discussion

Vaccination is the most effective way to prevent GCRV infection, and among the various vaccination methods, oral vaccine is the most valuable way to apply in aquaculture. For subunit oral vaccines, screening for suitable protective antigens is the key to developing subunit vaccines. In previous studies, the GCRV VP4 protein was bioinformatically analyzed, in which VP4-3 had the best antibody binding capacity and better protection rates (24), and the membrane protein of CyHV-2 was bioinformatically analyzed, in which the ORF25 protein had the best immunogenicity, and better protection rates could be obtained using a DNA vaccine encoding ORF25 (25). In our study, we verified that VP56-3 has the best antibody binding capacity and can produce more neutralizing antibodies to inhibit GCRV-II. Neutralizing antibodies are one of the indicators to evaluate viral vaccines (42), which suggests that VP56-3 is a potentially valuable antigen. Flagellin is a protein based Toll-like receptor agonist suitable as a multifunctional adjuvant for vaccines and immunotherapy and is widely used in mammals (33). The flagellin (FlaB) can upregulate TLR5 expression level, and vaccines with FlaB added as an adjuvant in mammals can more effectively enhance antibody response (43). However, at present, FlaB as an adjuvant has rarely been reported in aquatic viral vaccines. Based on the situation, we developed an oral microencapsulated vaccine based on VP56-3 protein and adjuvant FlaB delivered by sodium alginate, which provides a new strategy for large-scale application in aquaculture for the prevention of GCRV infection.

Sodium alginate delivery provides a new approach to overcome the disadvantages of protein, especially in terms of controlled release and avoidance of digestion by enzymes (23). The principle of sodium alginate encapsulation of drugs is the encapsulation of active substances by divalent cations or small molecule cross-linkers in a cross-linking process. In previous studies, sodium alginate capsules encapsulated with *Bacillus subtilis* were more effective in resisting the harsh environment of the stomach and intestines (14), encapsulated BSA microcapsules prepared using PLGA/alginate have good encapsulation rates and slow release properties (35). In our study, we successfully encapsulated VP56-3 antigen and FlaB adjuvant in sodium alginate to obtain oral microencapsulated vaccine SA-VP56-3/FlaB. SA-VP56-3/FlaB has excellent stability, biocompatibility, and non-toxicity. Previous studies have shown that proteins are readily hydrolyzed by proteases in the stomach and intestines, resulting in significant impairment of protein activity (44). In the observation of intestinal *in vivo* fluorescence, SA-VP56-3/FlaB can be present in the intestine for at least 12 h and reach the hindgut site, while the visualized fluorescence of the hindgut was significantly reduced in the free protein VP56-3/FlaB group at 12 h. The hindgut is the primary location where fish exert mucosal immune function (45, 46), suggesting that SA-VP56-3/FlaB are more likely than free VP56-3/FlaB to act *in vivo* to activate the mucosal immune barrier in the intestine. SA-VP56-3/FlaB also has sustained release properties for 24 h. Previous studies have shown that sustained release of protein can more effectively stimulate an immune response in the host (47).

Protection rates are the best means of assessing vaccines. In previous studies, in mice against listeriosis, oral vaccines encapsulated with sodium alginate improved protection rates by 60% relative to the control (36), in another experiment, oral inactivated vaccines were prepared with chitosan-aluminum improved protection rates by 47.4% relative to the control (34). In our experiments, the SA-VP56-3/FlaB group had the highest protection rate, which was 46% higher compared to the control group. These results suggested that SA-VP56-3/FlaB prepared with sodium alginate is an excellent oral vaccine against GCRV infection, and we subsequently explored the effects by oral administration with SA-VP56-3/FlaB on the host.

Antibodies play a crucial role in humoral immunity. Among the three immunoglobulins, IgM is considered to be the most abundant immunoglobulin in blood and mucosa associated tissues and is the most important immunoglobulin in systemic immunity for improving disease resistance in fish (48). IgZ is considered to play an important role in mucosal immunity, and it is ubiquitous in the intestine, skin, and gills (48, 49). In previous studies, most effective vaccines induced a high level of IgM, which is critical in determining the quality of the subsequent adaptive immune response (11, 39). In addition, effective oral vaccines can induce the production of IgZ in intestinal mucosal immunity (50). In our study, the expression level of IgM mRNA in the SA-VP56-3/FlaB group was significantly upregulated in the spleen and head kidney, and the expression level of IgZ mRNA was significantly upregulated in the hindgut. Upregulation of IgM mRNA suggests that B cells can be activated, and humoral immunity is effectively induced in the host (51), and upregulation of IgZ mRNA indicated that intestinal mucosal immunity was induced (50). The SA-VP56-3/FlaB group had the best serum binding to antigen and produced more neutralizing antibodies to inhibit GCRV-II on D28. Previous studies have shown that the concentration of antibodies usually corresponds to the protection/survival rate after vaccination (28, 52). In our study, the survival rates of the groups had a correspondence with the neutralizing antibody. This suggests that antibodies with neutralizing activity contribute to the host’s immune defense and inhibit virus transmission through systemic circulation (14). These results suggested that SA-VP56-3/FlaB can significantly promote humoral immunity and induce high levels of antibody production, resulting in better protection of grass carp.

In lower animals, their adaptive immune system has some limitations especially fish, such as their limited antibody pool and the slow proliferation, maturation, and memory processes of lymphocytes, therefore, non-specific immunity is also very important as the first line of defense against pathogenic invasion (37). Lysozyme (LZM) is an innate immune defense component of leukocytes that binds directly to negatively charged viral proteins and forms complexes with DNA, RNA, and deoxyribonucleoproteins to inactivate viruses (53). TSOD is a potent antioxidant that reduces intracellular levels of oxidant stress (54). The complement system can modulate the host immune by inducing innate immunity and regulating acquired immunity. C3 is the central molecule of the complement system and its activation is essential for all important functions performed by the system (39, 53). In our study, we found that LZM, TSOD, and C3 activities were significantly higher in the SA-VP56-3/FlaB group than those in the other groups. The increase in the activity of these indicators could be due to an increase in the number of cells involved in this process (e.g. migration of head kidney leukocytes) or enhanced pathogen resistance (53). This also suggested that the relatively low degradation of antigens in the SA-VP56-3/FlaB group may play an important role in nonspecific immunity. However, the exact mechanisms involved need to be further explored.

In addition, SA-VP56-3/FlaB significantly reduced the tissue viral load of GCRV-II in the head kidney, spleen, and hindgut. Therefore, it is also necessary to investigate the effect of SA-VP56-3/FlaB on the adaptive immune mechanisms of the organism. MHC molecule belongs to the immunoglobulin superfamily and is central in adaptive immunity, where they regulate specific B- and T-cell responses (55). MHC-I molecule can bind to the T-cell surface marker gene CD8 and mediate cytotoxic effects. Some studies have shown that tuna showed significant upregulation of the CD8 gene after inoculation with alloantigens (56, 57), and in zebrafish, the CD8 gene also showed significant upregulation after challenge (58). MHC-II molecule is mainly to deliver processed antigen fragments to CD4+ T cells during the initiation phase of the immune response and to modulate humoral immunity (59). In a previous report, it was shown that vaccination upregulated MHC-II mRNA expression level in the head kidney (60), and in another experiment the number of MHC-II positive cells was increased at the site of infection, suggesting that these cells enhance the immune response by antigen presentation capacity to induce antibody responses (61). In our study, both CD8 and MHC-II were significantly upregulated in the SA-VP56-3/FlaB group in the head kidney, spleen, and these results suggested that the protective effect of SA-VP56-3/FlaB might be due to the induction of humoral and cellular immune responses. Similar results were observed in other studies, where the expression levels of a series of genes MHC-I, MHC-II, CD4, and CD8 were significantly upregulated in the spleen of immunized grass carp (53, 62). IFN1 plays a crucial role in host defense against the virus (63), and it has been previously shown that GCRV recombinant VP35 protein could promote the induction of IFN-I and TLR22 expression in grass carp for defense against GCRV infection (64), and in our study, SA-VP56-3/FlaB can significantly upregulate IFN1 mRNA expression level in the head kidney and spleen, indicating that SA-VP56-3/FlaB can defend against GCRV infection.

## 5 Conclusion

We designed and prepared an oral microencapsulated vaccine (SA-VP56-3/FlaB) with excellent stability, low toxicity, and slow-release, which can effective against hydrolysis by proteases of the intestine. Oral immunization of SA-VP56-3/FlaB can induce an effective immune response, significantly alleviate tissue viral load and reduce mortality. This research has developed a promising oral vaccine that can be easily applied in aquaculture, providing a strategy for against GCRV-II infection in aquaculture.

## Data Availability Statement

The original contributions presented in the study are included in the article/**Supplementary Material**. Further inquiries can be directed to the corresponding author.

## Ethics Statement

All procedures of animal experiments were approved by the Ethical Committee on Animal Research at Huazhong Agricultural University (ID Number: HZAUFI-2021-0028).

## Author Contributions

JS and CX conceived and designed the experiments, and wrote the manuscript. CX carried out most experiments and data analyses. MQ and XH performed qRT-PCR assay. ZL expressed and purified proteins. JS revised the manuscript critically. All authors reviewed the manuscript. All authors contributed to the article and approved the submitted version.

## Funding

This work was supported by Fundamental Research Funds for the Central Universities (2662021SCPY006) and National Key Research and Development Program of China (2018YFD0900504).

## Conflict of Interest

The authors declare that the research was conducted in the absence of any commercial or financial relationships that could be construed as a potential conflict of interest.

## Publisher’s Note

All claims expressed in this article are solely those of the authors and do not necessarily represent those of their affiliated organizations, or those of the publisher, the editors and the reviewers. Any product that may be evaluated in this article, or claim that may be made by its manufacturer, is not guaranteed or endorsed by the publisher.
