# Supplementary material for: An Oral Microencapsulated Vaccine Loaded by Sodium Alginate Effectively Enhances Protection Against GCRV Infection in Grass Carp (Ctenopharyngodon idella)
Source: Front Immunol. 2022 Mar 24;13:848958. doi: 10.3389/fimmu.2022.848958 (PMC8987307; doi:10.3389/fimmu.2022.848958)
Supplement: Supplementary file 1 [file Table_1.docx]

# Supplementary material

## Table S1 Primer sequences in this study.

| Gene name | Primer direction | Primer sequence (5’-3’) | Size (bp) |
| --- | --- | --- | --- |
| VP56-1 | Forward | GCGGGATCCATGGCCACTCGTGACAGCC | 420 |
|  | Reverse | CCGCTCGAGTTAACGCACATCGTACCCGTT |  |
| VP56-2 | Forward | GCGGGATCCATGAGCTTTCCACCTATAGCCG | 522 |
|  | Reverse | CCGCTCGAGTTAGTTCAAAGTCAAATATCCGTCAA |  |
| VP56-3 | Forward | GCGGGATCCATGAGGTCCCAACGAAAATTG | 525 |
|  | Reverse | CCGCTCGAGTTATAAAATCAACTCGATGCCATCC |  |
| VP56 | Forward | CGCGGATCCATGGCCACTCGTGACAGC | 1539 |
|  | Reverse | CCGCTCGAGTTACTTACAGCAAACTACCGTCC |  |
| VP56-Q | Forward | GCACAACTAGCAGGCTATTCA | 115 |
|  | Reverse | GTTCTAACGCTCACCGTCTTT |  |
| IFN1 | Forward | GGTGAAGTTTCTTGCCCTGACCTTAG | 173 |
|  | Reverse | CCTTATGTGATGGCTGGTATCGGG |  |
| MHC-II | Forward | TACTACCAGATTCACTCGG | 111 |
|  | Reverse | CGGGTTCCAGTCAAAGAT |  |
| CD8 | Forward | GAGTCTCTGCACGGATCTAT | 172 |
|  | Reverse | GTGTAGTGTTCCGAATTTAAGT |  |
| IgM | Forward | TGGAGCAACGGCACAGTATT | 131 |
|  | Reverse | TCTGGGGGTGCTAACAGGTA |  |
| IgZ | Forward | TACATAGAGGGCAGAACCAG | 110 |
|  | Reverse | GTAACATTGGGTTTCCTTCC |  |
| 18S rRNA | Forward | ATTTCCGACACGGAGAGG | 90 |
|  | Reverse | CATGGGTTTAGGATACGCTC |  |
